# Stress, pseudoallergens, autoimmunity, infection and inflammation in chronic spontaneous urticaria

**DOI:** 10.1186/s13223-019-0372-z

**Published:** 2019-09-11

**Authors:** Ciara Jade Bansal, Amolak Singh Bansal

**Affiliations:** 1 St Barts, Queen Mary’s University, London, UK; 2 St Helier Hospital, Carshalton, Surrey SM5 1AA UK

**Keywords:** Chronic urticaria, Pseudoallergens, Stress, Infections, Autoimmunity, Cofactors, Vitamin D3

## Abstract

Chronic spontaneous urticaria (CSU) is often associated with organ specific autoimmunity but is rarely caused by food allergy. Colourings and preservatives in pre-packaged foods, so called pseudoallergens, have also been implicated. Factors that promote inflammation or reduce anti-inflammatory mechanisms may however, predispose susceptible individuals to CSU. Chronic underlying infection and mental and emotional stress can sometimes precede the onset of CSU and once established can exacerbate the symptoms. There is early evidence of dysbiosis within the gastrointestinal tract in people with CSU and reduced levels of vitamin D are also evident. The latter may be related to the importance of vitamin D3 in increasing T regulatory function which can control a tendency to autoimmunity. It is quite possible that a state of on-going chronic inflammation with reduced anti-oxidant mechanisms may underlie the not infrequent association between CSU and metabolic syndrome. Effective treatment of CSU should involve the use of anti-histamines, intermittent steroids and anti-IgE therapy. For recalcitrant disease immune modulatory therapy has a place. However, talking therapies that reduce stress and anxiety, vitamin D3 supplementation, correction of intestinal dysbiosis and treatment of any chronic infection should also be considered.

## Background

Chronic spontaneous urticaria (CSU) is generally defined as recurrent urticaria present continuously or intermittently for 6 or more weeks. Most cases of acute urticaria resolve—less than 8% will continue to CSU [1]. It is frequently a mast cell driven disease with clinical features arising from the action of several factors released by activated and degranulated mast cells. Indeed, mast cell numbers are increased three fold in the involved and uninvolved skin of patients with CSU [2]. It has been estimated to present a lifetime risk of 20% and with an annual prevalence of approximately 0.02 to 0.5% [3, 4]. CSU is more frequent in females with a male to female ratio varying between 1.5:1 to 2:1. Such female preponderance is evident in many of the organ specific and systemic autoimmune diseases [5]. While autoimmune disease (21%), chronic infection (29%) and immune dysfunction (4%) may become evident over time, CSU remains idiopathic in 45% even after 10 years of follow-up [6].

**Fig. 1:**
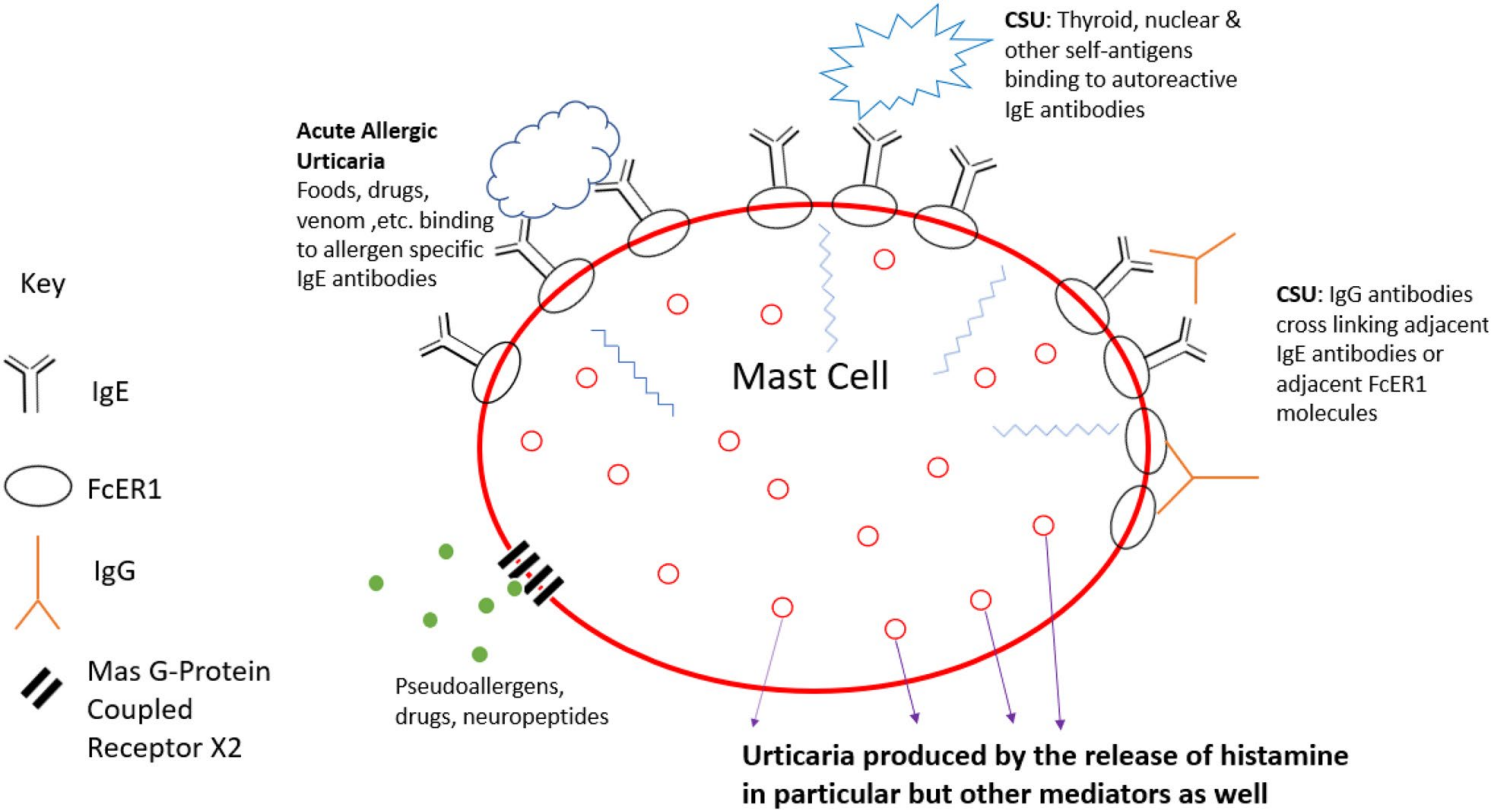
Mast cell activation in acute and chronic spontaneous Urticaria

Aetiologically, and despite the unequivocal benefit of anti-IgE therapy in severe CSU [7], IgE mediated type I food allergy is considered rare [8], although associated atopy is increased, especially in adolescents with CSU [9]. Much recent work has focused on other mechanisms leading to mast cell activation which is critical to CSU [7]. In particular, this includes autoimmunity type IIb with IgG anti-IgE auto-antibodies and positive autologous serum skin tests. Other mechanisms include type I IgE auto-antibodies directed at self-antigens, CD4-positive T cells directed at the high-affinity IgE receptor, immune cell activating IgE, and histamine-releasing factors able to bind to IgE. More recent work suggests a central importance of the Mas related G protein coupled X2 receptor (MRGPRX2) that is found on mast cells. This can be stimulated by the neuropeptides released by stress, defensins, pseudoallergens and various medications [10, 11]. This is summarised in Fig. 1.

CSU is associated with several disorders which either predispose to the condition or facilitate mast cell degranulation. Additionally, several conditions appear to frequently co-exist with CSU and appear to be linked by a common underlying pro-inflammatory state. These are summarised in Table 1. In many patients with CSU the unpredictability of the urticarial wheals and overwhelming nature of the itching can lead to persistent stress [12]. This is frequently associated with a significant alteration of mood and a markedly reduced quality of life [13, 14]. Each of these factors should be addressed in the investigation and management of patients with CSU. Certainly, efforts to reduce these variables may lessen the severity and duration of the condition with a lowered need to use second and third line therapies.

**Table 1:** Conditions associated with affecting the prevalence and severity of CSU

Variable	Contribution to CSU	Outline of purported mechanism
Autoimmunity	Predisposing factor	CSU is more frequent in females, associated with a positive autologous serum skin test and is frequently associated with underlying autoimmunity and altered T cells subsets. Diversely autoreactive IgE and IgG autoimmunity is particularly frequent in CSU. Benefit with anti-IgE therapy suggests direct ability of IgE auto-antibodies in triggering mast cell degranulation
Pseudoallergens	Facilitating factor	These low molecular weight compounds may bind to mast cell Mas related G protein coupled receptor X2 and lower the threshold for other factors to fully activate the mast cells to release CSU mediators. Salicyclates and non-steroidal anti-inflammatory drugs in predisposed individuals increase overall leukotriene activity by COX-1 inhibition. These then lead to mast cell activation and increased CSU activity
Stress	Facilitating or predisposing factor	Increased inflammation with altered T cell subsets and a reduction in Tregs especially leading to impaired B cell control. Stress released neuropeptides can also activate mast cells via Mas related G protein coupled receptor X2
Parasitic infection	Predisposing factor	Parasites stimulate humoral autoimmunity especially a polyclonal IgE which may have auto-reactive components
Helicobacter gastritis	Predisposing factor	While the frequency of helicobacter infection may be higher in CSU patients evidence of anti-helicobacter therapy being effective is conflicting
Metabolic syndrome	Co-morbid condition	Both CSU and metabolic syndrome are associated with increased background inflammation. As such the association between these may be due to the chronic inflammation being common to both
Hypertension	Co-morbid condition	CSU more likely to be prolonged in patients with hypertension; hazard ratio 0.71
Dysbiosis of gastrointestinal tract	Predisposing factor	Reductions in several types of bacteria in the stools of those with CSU but not enterobacteriaceae. Altered bowel microbiota may lead to increased gut epithelial permeability and absorption of immune activating compounds
Vitamin D3	Facilitating factor	Low levels found in CSU. Vitamin D3 reduces Th1 and Th17 cells and increases T regulatory cell function that can reduce autoimmunity and reduce inflammation

## Aetiological and associated factors in CSU

### Pseudoallergens

The importance of low molecular weight preservatives, colourings, salicylates and histamine in CSU has waxed and waned over the years. While salicylate mediated inhibition of cyclooxygenase 1 leading to a disturbance in a healthy prostaglandin/leukotriene ratio [15, 16] may underlie an exacerbation of CSU in some patients, the factors other than polymorphisms in centrosomal protein of 68 kDa [17] that predispose to this are unclear. Clearly, the small size of these molecules makes it impossible for them to directly bind IgE and there is no evidence that they act as haptens. Nevertheless, there is compelling evidence that a significant proportion of patients with CSU may benefit from a diet low in these ‘pseudoallergens’ [18–21]. This was associated with reduced leukotriene E4 excretion in the urine compared to pre-diet levels [18]. However, provocation testing with ‘known food dyes and preservatives’ was positive in only 18% of CSU patients although 73% had originally responded to the pseudoallergen free diet [22]. Interestingly, 71% of these patients reacted to provocation testing with pureed tomato and 41% to a steam extract to tomatoes. In a similar way, Rajan et al. [23] noted only 2 of their 100 patients with CSU reacted to a single blind challenge with 11 of the most frequently used additives. Finally, intestinal permeability was reported to be increased in CSU and this was partly reversed by 24 days on a reduced pseudoallergen diet [24]. Collectively, these results suggest that CSU may in some people be due to a temporary sensitivity to pseudoallergens and natural low molecular weight compounds which enter the body across a leaky gut epithelium. The pseudoallergens may in themselves also contribute to the leaky gut but it is likely that other factors may be responsible for raising mast cell sensitivity to the pseudoallergens. However, once these additional factors have subsided then normal resistance to these compounds may once again prevail.

### Autoimmunity

The presence of a positive autologous serum (AST) or plasma test (APT) in 40% to 60% of patients with CSU has suggested the presence of auto-antibodies capable of stimulating mast cells [25]. Importantly, a positive AST has been shown to increase the possibility of acute spontaneous urticaria progressing to CSU [26] and a reduced chance of resolution within 2 years [27].

Several types of autoimmune diseases are increased in patients with CSU (Table 2). These include particularly hypo and hyperthyroidism but also diabetes, rheumatoid arthritis and Sjogren’s syndrome [28]. IgG anti-thyroid antibodies, in particular, are not infrequent in patients with CSU [29] and especially in females [30]. However, IgE antibodies to TPO have also been detected [31, 32] suggesting a possible mode of action for the benefit of anti-IgE therapy in CSU. Interesting recent work also suggests that a high proportion of the total IgE in those with CSU may be autoreactive and have increased lipophilicity [33]. Regardless, IgG antibodies directly binding to the FcεRI and in consequence capable of activating mast cells have been and found in almost a quarter of patients with CSU with only 3.2% in healthy controls [34]. This has kindled interest in mast cell activating IgE and IgG autoantibodies directed to thyroid and nuclear antigens. Interestingly, passive transfer of IgE anti-TPO antibodies could elicit positive skin prick test response in control subjects and basophils from patients with CSU and anti-TPO antibodies could be activated by incubation with TPO [35]. At a more basic level, auto-antibodies in general may encourage the secretion of Type I interferons by plasmacytoid dendritic cells (pDC) [36] and stimulate recruitment of basophils to lymph nodes and directly activate adaptive immune responses through B and T cells [37]. In an interesting recent report IgE antibodies to IL24 functionally capable of activating mast cells and correlating with disease activity have been reported by Schmetzer et al. [38]. The authors also noted IgE auto-antibodies to over 200 self-proteins from a total of over 9000 proteins screened. The avidity of these antibodies remains unclear and it is possible that they represent non-specifically auto-reactive antibodies generated as part of a polyclonal humoral response to a viral or other infection.

**Table 2:** Frequency of various autoimmune diseases in CSU

Autoimmune condition	Level of risk
Hashimoto’s thyroiditis	Overt hypothyroidism about 5% although anti-TPO abs can be found between 10 and 20% This figure varies between studies
Pernicious anaemia	5%
Grave’s disease	5%
Vitiligo	5%
Insulin dependent diabetes	> 1%
Coeliac disease	> 1%
Rheumatoid arthritis	> 1%
Polyglandular syndrome with autoimmune thyroid disease, pernicious anaemia and or vitiligo	> 1%
Systemic connective tissue disorders e.g. lupus, MCTD etc.	Same as background population prevalence

Clinically the increased prevalence of autoimmunity in CSU suggests impaired immune regulation and an imbalance in the ratio of the pro-inflammatory Th17 cells and T regulatory cells (Tregs). Tregs dysfunction, and especially T follicular regulatory cells [39], leading to the survival of autoreactive T and B cells and altered antigen presenting function by dendritic cells may underlie several systemic and organ specific autoimmune diseases [40, 41]. In this regard, it is noteworthy that impaired T regulatory (Treg) cell function has been found in CSU [42, 43] with alterations in other T cell subsets also being evident [44]. From a therapeutic perspective efforts to improve T reg function may therefore be helpful to reduce the severity and duration of CSU. At present this has been utilised predominantly in graft versus host disease after hematopoietic stem cell transplantation [45].

### Infections

While the precise cause of CSU is often unclear in an individual patient, viral infections and increased stress levels may be important factors in Western countries [46]. Globally, however, parasitic infections may be of greater importance with an average comorbidity of around 10% [47]. In this group anisakis, toxocariasis, fasciolosis, strongyloidiasis and blastocystosis were the most frequent parasitic infections and treatment led to improvement in an average of one-third of patients across several studies [47]. In this regard, empirical anti-helminth therapy was suggested by Nahshoni et al. [48] for people with CSU returning from travel to ‘developing countries’. Earlier work by Hameed et al. [49] suggested that the urticaria could be more properly linked with the amoeboid form of blastocystis hominis. Furthermore, 60% of these patients responded to a single course of metronidazole and 100% to two courses.

More recent work has shown an increased frequency of dysbiosis of the gastrointestinal tract in CSU. As such there was a significantly reduced frequency of *Akkermansia muciniphila*, *Clostridium leptum* and *Faecalibacterium prausnitzii* in the stools of CSU patients while that of Enterobacteriaceae was unchanged [50]. Intriguingly, the number of tryptase and CD117 positive mast cells per high power field were significantly higher in patients with CSU compared to controls and adjunctive treatment with mast cell stabilisers has been suggested [51].

In regards to the role of helicobacter infection the precise significance of this infection in precipitating and/or aggravating CSU has oscillated over the years. More recent work suggests that asymptomatic infection is more frequent in CSU patients than in healthy controls and in helicobacter infection positive patients the severity and extent of the urticaria may be more severe [52].

In terms of CSU and infections outside of the GI tract, there are numerous anecdotal reports or small case series that have purported to show a link with viral, bacterial or fungal infections. Thus, Dreyfus [53] noted serological evidence of human herpes virus 6 infection, sometimes in association with Epstein Barr virus reactivation, to be more frequent in patients with CSU. In their systematic review, Imbalzano et al. [54] considered herpes viruses to be possible aetiologic factors in children and hepatitis viruses were more frequently associated with CSU in adults. The precise role of so-called ‘superallergens’ that can non-specifically stimulate basophils and mast cells and which are produced in viral infections such as hepatitis C and HIV is unclear [55].

In the case of bacterial infections, Calado et al. [56] found streptococcal tonsillar infection to relate to CSU with improvement after antibiotic therapy and more permanent resolution after tonsillectomy. Godse and Zawar [57] found a link with tinea pedis in four patients with significant improvement after anti-fungal therapy. However, Cribier and Noacco [58] did not support a definite link between focal infections such as sinusitis and dental infections and CSU. They also mentioned the importance of curative antibiotic therapy in eliminating CSU as clearly supporting an aetiological link and this was frequently absent in the case of focal infections.

The mechanism by which infection contributes to the onset, perpetuation or worsening of CSU is unclear. It is possible that different components of the immune response to infection may be responsible in varying combinations to these different stages in CSU. As such Kay et al. [59] reported increased cellular expression of the Th2 cytokines IL4 and IL5 as well as IL33, IL25 and thymic stromal lymphopoietin in the lesional but not non-lesional skin of patients with CSU. Notably, the IL25 and IL33 was expressed by multiple cells involved in the innate immune system. In the case of helicobacter infection, a 23 to 35 kDa protein obtained from helicobacter preparations was found to induce the release of histamine, TNF-a, IL-3, IFN-γ, and leukotriene B4 by the LAD2 mast cell line in a dose or time-dependent manner [60]. Additionally, mast cells can be activated by inflammatory cytokines such as IL6 [61] and raised levels of IL6, IL1β and TNFα have been reported in patients with infection and in CSU [62]. It is therefore not inconceivable that infections associated with inflammation may be associated with urticaria. Indeed, some patients with CSU have been improved by the discovery and treatment of occult infection [63]. Overall, however, it is likely that infection acts as a facilitating factor for the initiation and perpetuation of CSU and additional cofactors such as stress may be required for the CSU phenotype to be expressed. This would explain why few patients with severe infections such as a pneumonia, pyelonephritis, abscess etc. develop urticaria.

### Stress and altered mood

Urticaria has been reported to be increased in frequency in patients with bipolar disorder [64] which is well known to be accompanied by high levels of stress. CSU is also associated with a significantly higher prevalence of depression, anxiety and poor quality of sleep [3, 65]. In the recent report by Tat [66], depression and anxiety assessed using the Hospital Anxiety and Depression scale was evident in almost half of the 50 CSU patients seen and with a significant positive correlation of both with the urticaria activity score. Regardless, the coexistence of mood disturbance with CSU can have major repercussions for work attendance and efficiency [3, 14]. In children, high rates of various sorts of anxiety as well as social phobia and depression have also been reported [67]. Interestingly, ‘state anger’ measured using the State trait anger expression inventory correlated significantly with levels of pruritis [68]. High levels of anger were also reported by Altınöz et al. [69]. Overall, there is increasing evidence that continued stress can perpetuate and aggravate CSU.

As mentioned previously, patients with CSU have increased levels of emotional distress with an underlying anxiety, depression and somatoform disorder [12]. Conversely, increased levels of stress may perhaps predispose individuals to CSU. As such Yang et al. [70] noted significantly more ‘life events, higher subjective weighting of impacts from life events, more somatic symptoms, more severe insomnia, less family support and more negative coping tendencies’ in 75 consecutively seen CSU patients than 133 control patients with tinea pedis. However, the mechanism by which stress predisposes to CSU is unclear. Interestingly, the basophils of patients with CSU appear to activate more readily with adrenocorticotrophic hormone and corticotrophin releasing factor and to have had a trend to raised cortisol levels compared to healthy controls [71]. Additionally, in mice, stress can lead to a disruption of T regulatory cell function with a shift towards a Th1/Th17 balance leading to exaggerated autoimmunity [72]. In children, stress has been associated with an increased tendency to autoreactive T and B cells and with the production of IgG auto-antibodies [73]. Increased activation of the NLRP-3 inflammasome has also been demonstrated in major depression and stress and this may provide the link between psychological factors and exacerbation of urticaria by changes in mood and especially emotional stress [74].

The link between the brain and mast cells within the skin via C fibre sensory nerves is likely involved in the worsening of skin diseases such as atopic dermatitis and CSU with stress [75]. The increased frequency of CSU in women is likely related to oestrogen and progesterone and the ability of these hormones to stimulate autoimmunity [5]. However, recent work also suggests the importance of pituitary adenylate cyclase activating polypeptide in stress circuits and stress responses and which is modulated by oestrogen [76]. In terms of the chemical link between nerves and mast cells, several neuropeptides, but particularly substance P are able to activate mast cells [77] leading to the release of histamine and related mediators that are involved in CSU. Interestingly neuropeptides such as vasoactive intestinal peptide, α-melanocyte-stimulating hormone and calcitonin gene-related peptide (CGRP) are evident both within the central nervous system and released by cutaneous nerves [78, 79]. In this regards it is noteworthy that stress leading to the release of sensory nerve neuropeptides can alter the behaviour of Langerhans cells within the dermis and skew the cutaneous immune system towards specific T helper cell pathways [80]. CGRP, in particular, appears to encourage Th17 type cells with the potential to increase inflammation by the recruitment of T cells and neutrophils [78, 81].

Recent work suggests that stress induced release of substance P and CGRP by sensory cutaneous nerves leads to itching and mast cell activation via several receptors. These include the Mas-related G protein-coupled receptors (Mrgpr) family as well as transient receptor potential ankyrin 1 (TRPA1) and protease activated receptor 2 (Par2) [78]. The subsequent release of cytokines such as IFN-γ, IL-4, TNF-α, and IL-10 then encourages immune activation and the aggravation of cutaneous inflammation. It is therefore possible that the itch scratch cycle can prolong the cutaneous inflammation that perpetuates CSU and which is also seen in atopic dermatitis.

### Chronic inflammation, oxidative stress and metabolic syndrome

Many patients with CSU are observed to have mildly raised levels of C-reactive protein. Determining the cause of this abnormality is frequently unrewarding. However, a state of immune readiness and inflammation has been suggested to be present in the peripheral blood of CSU patients by Santos et al. [82]. They reported significantly higher serum levels of several chemokines (CXCL8, CXCL9, CXCL10 and CCL2) in patients with CSU compared to healthy controls and this was irrespective of whether they were ASST positive. Furthermore, the basal secretion of CCL2 by peripheral blood mononuclear cells or induced by Staphylococcus aureus enterotoxin A was higher in those with CSU as indeed was CXCL8 and CCL5 secretion after phytohaemagglutinin stimulation.

In terms of oxidant stress, this has been found to be increased in children with CSU in whom total anti-oxidant activity was also reduced [83]. As such it is interesting that patients with CSU had lower levels of basal cortisol [84] and dehydroepiandrosterone-S (DHEA-S) [85]. Both are reduced in chronic stress and DHEA-S is partly regulated by the nervous system and is known to regulate the immune system. Furthermore, patients with CSU have an increased overall oxidative burden with reduced levels of erythrocyte copper-zinc superoxide dismutase (Cu-ZnSOD) levels [86]. This contrasts the situation in acute urticaria in which increased levels of oxidative stress indicated by raised levels of malonaldehyde and nitrous oxide were associated with increased Cu-ZnSOD and decreased levels of glutathione peroxidase [87].

Chronic low grade inflammation associated with high stress scores, raised C-reactive protein and interleukin 18 [84] may explain the increased frequency of metabolic syndrome in CSU [88, 89]. Hypertension was noted in 18.1% of the 1539 CSU patients that were analysed as part of the German contribution to the AWARE study [90]. The mechanism by which hypertension may prolong CSU is, however, unclear [91] although it should be borne in mind that ACE inhibitors used in hypertension can aggravate and prolong CSU.

### Vitamin D3

Vitamin D3 (cholecalciferol) has a powerful ability to increase T regulatory cell function and reduce Th1 and Th17 type immunity [92]. These immune changes can help ameliorate autoimmunity. In this regard, it is interesting that reduced levels of serum vitamin D3 have been found in several of the systemic autoimmune diseases as well as in asthma and inflammatory bowel disease. In CSU, reduced vitamin D levels are found more frequently than in healthy controls [93] and replacement therapy has been found to reduce the severity and duration of the urticarial wheals [94, 95].

### Coagulation pathways

The involvement of the coagulation system in CSU is suggested by the clinical improvement in urticaria when patients are commenced on anticoagulation for other apparently unconnected reasons. Indeed, the present authors have seen this in 5 patients over the years. This is supported by the elevated d-dimer noted by Triwongwaranat et al. [96] in 58 out of 120 patients with CSU and with a correlation between d-dimer levels and severity of urticaria. Prior to this Sakurai et al. [97] observed increased thrombin generation by the peripheral blood cells of patients with CSU compared to healthy controls. In this respect, it is interesting that thrombin injected into the ear skin of mice produced a dose dependent degranulation of mast cells and the mast cells expressed thrombin receptors PARs 1, 3 and 4 [98]. Elevation of other coagulation variables has also been observed by other authors and treatment with anti-coagulant drugs as adjunctive therapy suggested [99]. More recently Yanase et al. [100] have suggested the involvement of the extrinsic coagulation pathway in CSU and highlighted the role of tissue factor expression of endothelial cells as being important. Notwithstanding, the precise mechanism by which coagulation contributes to urticaria is unclear although the interaction between inflammatory factors and coagulation proteins is interesting [101].

### Altered gut permeability

Patients with coeliac disease have been shown to have an increased frequency [28] and susceptibility to both acute and chronic urticaria with odds ratios of 1.31; 95% CI 1.12–1.52 and 1.54; 95% CI 1.08–2.18, respectively [102]. The mechanism here is unclear but as coeliac disease is increased in those with IgA deficiency and the latter is associated with autoimmunity this may represent one possible mechanism. Additionally, coeliac disease is associated increased permeability of the upper gastrointestinal tract which may allow the greater absorption of pseudoallergens. Furthermore, vitamin D deficiency is more frequent in coeliac disease which may in turn contribute to impaired T regulatory cell function and raised tendency to autoimmunity.

## Linking the different factors that facilitate the onset of CSU

The ability of pseudoallergens, infections and stress to initiate and/or aggravate CSU suggests that a common pathway may be involved. However, in some patients with CSU type 1 and 2a immune hypersensitivity with specific IgE and autoreactive IgG antibodies may be directly involved in activating mast cells. In this case the condition continues until the respective IgE and autoreactive B cells are lost or the antigenic stimulus is eliminated. However, an increased frequency of autoimmunity has been linked to stress [5, 73, 103–106] and several viral infections, but especially Epstein Barr, can encourage autoimmunity [107, 108]. The mechanisms here involve a combination of impaired T regulatory activity and specific viral proto-oncogenes that give a survival stimulus to low avidity auto-reactive B cells. These would normally suffer spontaneous demise or be actively eliminated. Consequently, certain infections and stress may be linked to an increase susceptibility to CSU by perturbation of the immune system.

In the case of factors that facilitate the development of CSU, the role of the Mas related G protein coupled X2 receptor (MRGPRX2) found on mast cells may be relevant. Thus, increased expression has been found on the mast cells of patients with CSU compared to healthy controls [109]. Importantly, MRGPX2, previously known as MrgX2, can be stimulated by antimicrobial host defense peptides such as β-defensins and the cathelicidin LL-37 [110], neuropeptides such as substance P [109] and vasoactive intestinal peptide, major basic protein, eosinophil peroxidase, and several peptidergic drugs [10, 11]. Importantly, these multiple means of mast cell activation open the possibility that simultaneous subthreshold stimulation by several factors may cumulatively leading to mast cell degranulation and CSU. Thus, infection associated with host immune cell secreted defence peptides occurring during a period of stress and with an increased ingestion of foods with preservatives and colourings may together lead to urticaria whereas these factors alone may be insufficient. Moreover, the unpredictable continuation of the urticaria associated with raised stress levels may then perpetuate the CSU even when the original infection has been eliminated and there has been an improvement in the diet.

Varying degrees and patterns of inflammation are evident in CSU. In some there is a typical IgE mediated response with only low level lymphocytic and eosinophilic infiltration. In other cases, there is a greater involvement of neutrophils and mononuclear cells perhaps suggesting raised Th17 function. The pro-inflammatory cytokines mediating the latter type of immune response if unchecked for a prolonged period would likely aggravate and perpetuate CSU by encouraging inflammatory cell infiltration. However, the situation is rendered more complex by the ability of TNF-α to increase Tregs [45] and the fact there are several types of Tregs with slightly differing functions and ability to reduce autoimmunity [111].

## Therapeutic relevance of autoimmunity, pseudoallergens, stress, low vitamin D, infection and increased tendency to coagulation in CSU

**Fig. 2:**
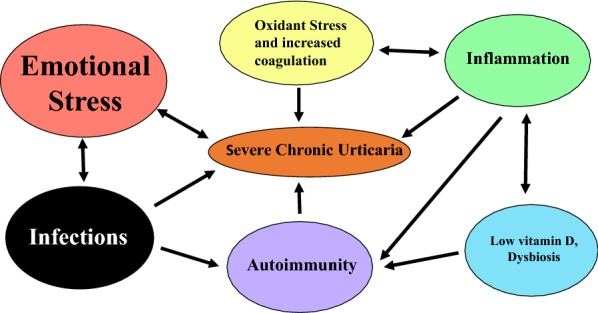
Interaction of different factors in Chronic Spontaneous Urticaria

The interaction of the more important factors that impact on CSU are shown in Fig. 2. Here it can be seen that many of the factors are interlinked and can directly or indirectly exacerbate CSU. Attention to each of these areas should form the basis of the holistic treatment of all patients with CSU. Regardless, most patients with CSU controlled by conventional dose second generation anti-histamine therapy do not require investigation according to the European and World Allergy Organisation guidelines on urticaria [112]. However, those unresponsive to high dose antihistamine therapy used up to quadruple levels may benefit from investigation for underlying autoimmunity, hypovitaminosis D, gastrointestinal dysbiosis and possibly helicobacter infection [113] depending on their clinical evaluation. Appropriate therapy should then be offered including vitamin D3, probiotics, anti-microbials and levothyroxine. The latter should certainly be offered if hypothyroidism is discovered [114]. The use of colouring and preservative free diets remains controversial but worth considering on a temporary basis in those patients who regularly consume pre-packaged food.

It has previously been shown that desloratadine can reduce oxidative damage to erythrocytes by increasing the activity of superoxide dismutase [115]. Thus, at least some anti-histamine therapies may work by both reducing the activity of histamine released by activated mast cells but also increasing anti-oxidative pathways that reduce inflammation. However, it is unclear whether oral anti-oxidant therapy provides any benefit to CSU patients on maximal anti-histamine therapy. Regardless, it should undoubtedly be considered in those with obvious poor diet and agents such as vitamin E and N-acetyl cysteine with their very low side effect profiles are ideal.

In rare reports, anti-coagulants have also been reported to be helpful in CSU patients with severe disease [116]. Although some have suggested that full carefully monitored anti-coagulation is required for efficacy [117] this is not always so. Indeed, some patients simply require low dose anti-coagulation that does not necessarily increase the prothrombin time or international normalised ratio. Indeed, low dose warfarin therapy was used with significant anecdotal benefit in the late 1980s.

As stress is a common precipitating or perpetuating factor in virtually all patients with CSU [118], efforts to uncover any obvious sources is important [12]. As such stress reduction therapy using a variety of approaches [119] may be helpful in improving CSU and should be considered as an important and early intervention to limit morbidity and improve overall quality of life. Interestingly, there is one report on the positive value of hypnotherapy in CSU although the benefit was most evident in those individuals who were considered hypnotizable [120].

At present anti-IgE therapy is recommended as third line treatment of severe CSU unresponsive to combinations of high dose anti-histamines [112]. The efficacy of additional leukotriene receptor antagonist therapy is unclear. Anti-IgE therapy appears at present to have very few short and long term side effects and is often better tolerated ciclosporin. There is also some evidence that it can normalise abnormal gene transcript signatures in lesional skin [121]. For the future, efforts to increase Tregs function and reduce Th17 activity would appear appropriate. This would be especially so in patients with severe CSU unresponsive even to anti-IgE therapy and perhaps especially in those with a positive autologous skin tests.

## Conclusion

The threshold for developing CSU is reduced by several common conditions while others frequently modify the condition or prolong its duration. These conditions are associated with impaired immune regulation and/or an underlying pro-inflammatory state and contribute to mast cell irritability and an increasing tendency to autoimmunity. The latter is often directed towards IgG and IgE cell surface receptors. Persistent stress and mild underlying infection is frequently evident in CSU and can activate mast cells via several neuropeptides and anti-microbial host defence proteins acting through the MRGPRX2. All factors known to affect urticaria should be addressed in the investigation and management of this condition. This may lessen the severity and duration of the CSU with a lowered need to use second and third line therapies.

## Data Availability

Data sharing is not applicable to this article as no datasets were generated or analysed during the current study.

## Abbreviations

CGRP: calcitonin gene-related peptide
CSU: chronic spontaneous urticaria
DHEA-S: dehydroepiandrosterone-S
IL: interleukin
IFN: interferon
MRGPRX2: Mas related G protein coupled X2 receptor
Th: T helper cells
TNF: tumour necrosis factor
Tregs: T regulatory cells
